# Assessment of Wood Bio-Concrete Properties Modified with Silane–Siloxane

**DOI:** 10.3390/ma16186105

**Published:** 2023-09-07

**Authors:** Amanda L. D. de Aguiar, Nathalia A. da Silva, Bruno M. C. Gomes, M’hamed Y. R. da Gloria, Nicole P. Hasparyk, Romildo D. Toledo Filho

**Affiliations:** 1 Department of Civil Engineering, COPPE, Federal University of Rio de Janeiro, Rio de Janeiro 21941-972, Brazil; amanda.aguiar@numats.coc.ufrj.br (A.L.D.d.A.); nathalia.silva@coc.ufrj.br (N.A.d.S.); bruno.gomes@numats.coc.ufrj.br (B.M.C.G.); yassin@numats.coc.ufrj.br (M.Y.R.d.G.); 2 Department of Dam Safety and Technology, ELETROBRAS Furnas, Aparecida de Goiânia 74993-600, Brazil; nicole@furnas.com.br

**Keywords:** wood bio-concrete, silane–siloxane, bio-aggregates, water absorption, durability

## Abstract

Bio-based materials, such as wood bio-concrete (WBC), hold promise in reducing energy consumption and carbon footprint of the construction industry. However, the durability of these materials is not well understood and can be negatively affected by the high water absorption capacity of wood bio-aggregates. In the field of cement composites, for example, silane–siloxane-based water repellent has been used to protect such materials from natural environmental attack. Nevertheless, there is still a limited understanding of various aspects related to this type of treatment, including its performance when applied to the bio-concrete substrate. This research aimed to investigate the influence of silane–siloxane on the rheology and hydration of cementitious paste through isothermal calorimetry and thermogravimetric analysis. Additionally, the impact of silane–siloxane on the physical and mechanical properties of WBCs was examined by conducting tests at fresh state (flow table and entrained air content) and hardened state (compressive strength and capillary water absorption). The composites were produced with a volumetric fraction of 45% of wood shavings while the cement matrix consisted of a combination of cement, rice husk ash, and fly ash. Silane–siloxane was applied in three ways: as coating, incorporated as an admixture, and in a combination of both methods. The results indicated that by incorporating silane in the cementitious pastethe viscosity increased by 40% and the hydration was delayed by approximately 6 h when compared to the reference. In addition, silane improved the compressive strength of WBCs by 24% when incorporated into the mixture, expressively reduced the water sorptivity of WBCs (93%), and was more effective if used as coating.

## 1. Introduction

One of the primary contributors to global carbon dioxide (CO_2_) emissions is excessive energy consumption [1]. Buildings account for over 40% of global energy consumption, corresponding to approximately 30% of the total CO_2_ emissions worldwide [2]. According to Boehm et al. [3], the construction sector can significantly reduce emissions by implementing four key changes: minimizing excess floor area, reducing energy intensity, decreasing emissions from energy use, and reducing embodied emissions in construction. As an alternative to reducing the amount of energy spent in civil construction, researchers are developing new energy-efficient materials. Thus, plant residues from industries and agriculture are being utilized in the production of sustainable insulation materials and as aggregates in concrete [4]. Another critical concern relates to the consumption of natural aggregates such as sand and gravel, which are globally the most extracted materials and face challenges regarding consumption regulation, leading to overexploitation and environmental degradation [5,6].

In this context, various plant residues are being utilized in the production of ecological concrete, including bamboo, hemp, rice straw, cotton, and wood-based materials [7]. The incorporation of bio-aggregates in cementitious composites has garnered significant attention as a means to mitigate CO_2_ emissions due to the biogenic carbon storage capacity of these aggregates [8]. Additionally, bio-concretes exhibit promising thermal properties, such as low thermal conductivity and high heat capacity, while also demonstrating satisfactory performance in terms of hygric regulation [9].

The annual global production of wood-derived biomass amounts to approximately 4.6 gigatonnes (Gt). Out of this total, around 60% is utilized for power generation, 20% is allocated to the “roundwood” industry, and the remaining 20% represents primary production losses that remain unused in the field [10]. Consequently, the utilization of wood waste as a valuable component in bio-concrete production has emerged as a means to mitigate environmental impact [11,12,13].

While bio-concrete with high biomass content holds significant potential for the construction industry, one major concern lies in the material’s durability. The deterioration of cementitious composites poses a significant problem as it can compromise the stability of the material. As wood is a heterogeneous and anisotropic material, it exhibits porosity that can lead to dimensional variations when exposed to external climatic conditions, although its benefits for thermal and acoustic dissipation arewell known. Consequently, this can impact the physical and mechanical properties of the produced bio-concrete. Factors such as relative humidity, temperature, water presence, and UV radiation induce changes in the chemical and microstructural properties of bio-based materials, thereby modifying their functional properties [14]. Water, in particular, poses a critical issue as wood particles are hydrophilic, and its presence results in the swelling of bio-aggregates, which increases tensions at the interface between plant particles and the binder [15].

To enhance the durability of concrete structures, various surface treatments can be employed. One effective method is the application of hydrophobic impregnations, which confer a water-repellent property to the concrete surface without significantly filling the pores. Silane-based products, particularly a mixture of silane and siloxane, are often preferred for their favorable water-repellent characteristics and good breathability [16]. These products have found wide usage in civil engineering applications [17,18,19]. They can be applied as an external surface protection measure [19] or incorporated as an admixture into fresh concrete during the production process [20], thereby imparting internal hydrophobicity to the matrix.

Recent studies have explored the use of silane as a treatment for wood wastes to enhance the properties of wood–cement composites [1,21,22,23]. Hyder et al. [1] were pioneers investigating the effect of two distinct silanes on wood composites to enhance compatibility between date palm wood fibers (DPWF) and polylactic acid (PLA), aiming to produce biodegradable composites. The treated composites exhibited significant improvements in mechanical properties and water resistance compared to untreated ones. Notably, the tensile strength of composites containing 20% PLA-UTDPWF increased remarkably from 8.6 MPa to up to 22.4 MPa. Aguiar et al. [21] explored the impact of accelerated aging in the laboratory on wood bio-concretes, using a silane-based water repellent as surface protection (SSP) and bio-aggregate impregnation agent (IBA). Both control and IBA samples exhibited reduced compressive strength post accelerated aging. The SSP exhibited enhanced capillary water absorption resistance during initial hours while IBA samples showed a 17% reduction compared to control at the end of the test.

Liu et al. [23] investigated the enhancement of wood–cement composites through the utilization of alkaline cooking (2–8% mass concentration) and silane coupling agent (1–5%) to modify wood chips. Alkaline cooking improved crystallinity and surface roughness while the silane agent enhanced interfacial properties. Compositions with 3% silane agent and 4% alkaline cooking exhibited an 11.9% increase in flexural strength compared to those with alkaline cooking only. The modification of wood chips through these methods demonstrated mechanical property improvements.

However, the influence of this water repellent when applied to bio-concrete is unknown. In this study, three different treatments involving silane–siloxane were investigated for wood bio-concrete: external coating, the incorporation of silane as an admixture, and a combination of both methods. To assess the physical and mechanical performance of the bio-concrete modified with silane–siloxane, several tests were conducted, including flow table test, entrained air content test, capillary water absorption test, and compressive strength test. The performance of the modified bio-concrete was compared with that of the reference mix. Additionally, the impact of incorporating silane–siloxane on the rheology and hydration of the cementitious paste was examined through isothermal calorimetry and thermogravimetric analysis.

The importance of this study lay in its capacity to address several essential factors. First, incorporating wood waste into bio-concrete helps reduce CO_2_ emissions through the biomass content in the mixture [24,25]. Furthermore, employing silane–siloxane treatment on wood bio-concrete holds promise for enhancing short-term material properties and ensuring long-lasting performance.

## 2. Materials and Methods

### 2.1. Materials

In this research, the cementitious materials employed included Lafarge-Holcim Portland Cement type CP II-F-40 (Rio de Janeiro, RJ, Brazil), rice husk ash from Silcca Nobre (Embu das Artes, SP, Brazil), and fly ash supplied by Pozo Fly Comércio de Cinzas Lima LTDA (Capivari de Baixo, SC, Brazil), with corresponding specific masses of 2970 kg/m^3^, 2250 kg/m^3^, and 1920 kg/m^3^, respectively. The specific mass measurements were conducted using a helium gas pycnometer, specifically the AccuPyc 1340 model manufactured by Micromeritics (Norcross, GA, USA).

**Figure 1:**
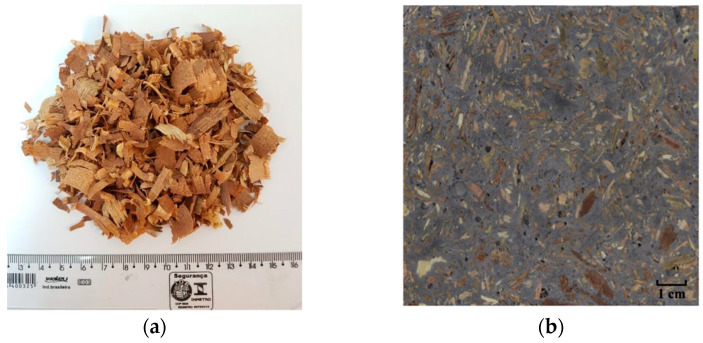
(**a**) Wood shavings; (**b**) wood bio-concrete.

The wood shavings utilized as bio-aggregates (Figure 1a) were obtained from a wood processing company located in the city of Rio de Janeiro, Brazil. These shavings were derived from four different wood species: *Hymenolobium petraeum*, *Cedrela fissilis*, *Erisma uncinatum warm*, and *Manilkara salzmanni*. To obtain suitable bio-aggregates for the production of bio-concrete, the wood shavings underwent a series of processes. Initially, coarse and fine particles were separated using a mechanical sieve. The fraction retained on the 1.18 mm sieve was selected for further use. Subsequently, the material underwent an alkaline treatment to eliminate extractives that could potentially hinder or inhibit cement hydration. The treatment involved immersing the wood shavings in a calcium hydroxide (Ca(OH)_2_) solution with a concentration of 1.85 g/L. The bio-aggregate-to-water ratio during the two-hour immersion was maintained at 1/10 and the drying of the bio-aggregate was carried out in ambient air for approximately 72 h [21]. The wood shavings were characterized by an apparent bulk density of 530 kg/m^3^ [26], moisture content of 19% [27], and water absorption of 70% as per the method proposed by Da Gloria and Toledo Filho [28].

The silane–siloxane-based water repellent used in this research, produced by Souza Filho Impermeabilizantes (Sorocaba, SP, Brazil), was fireproof and had low volatile emissions. It was composed of an aqueous anionic emulsion of isooctyl triethoxysilane. The density of the water repellent was approximately 1.01 ± 0.02 kg/L at a temperature of 25 °C.

### 2.2. Wood Bio-Concrete

The dosage of the bio-concrete in this study was determined using the rational mix design process developed by Da Gloria et al. [28]. This process was specifically designed for developing cementitious composites using bio-aggregates such as wood, bamboo, and rice husk. The dosage of the bio-concrete was established to minimize cement consumption, reduce the carbon footprint, and meet the required workability for molding through mechanical vibration. Drawing from previous research conducted on wood and bamboo bio-concrete [21,29], a volumetric fraction of 45% of bio-aggregate (BA) was selected in the composition. This choice was made considering the potential applications of the bio-concrete produced, such as in wall cladding, partition blocks, and retrofit panels for facades.

The cement matrix was composed, based on mass, of 45% Portland Cement (PC), 25% Rice Husk Ash (RHA), and 30% Fly Ash (FA), as used in previous works [21,29]. The water-to-binder ratio was set at 0.30, taking into account the hydration water (Wh). Next, an additional water named compensating water (Wc) was used based on the BA water absorption. A setting accelerator, calcium chloride (CC), was incorporated at a content of 2% relative to the mass of the cementitious materials, following the findings of previous study [30]. The proportion of the mixture produced in this research is summarized in Table 1.

The production process of bio-concrete involved the following steps: First, the wood shavings were mixed with the cementitious materials for 1 min. Subsequently, the total water that was previously mixed with calcium chloride was gradually added over a period of 1 min. To ensure complete homogenization, the overall mixing time was set at 4 min. The specimens were then cast in cylindrical molds with dimensions of 50 mm × 100 mm (diameter × height) in three layers. Each layer was subjected to mechanical vibration at a frequency of 68 Hz for 10 s. After 24 h, the samples were demolded and stored under dry curing conditions in a room with a relative humidity of 55 ± 5% and a temperature of 21 ± 2 °C until reaching the desired testing age. Figure 1b illustrates the visual appearance of the WBC45 specimens.

### 2.3. Silane Treatment Procedures

**Figure 2:**
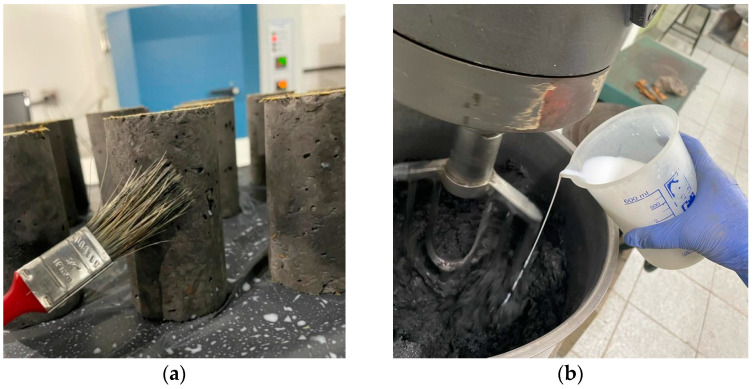
Silane treatments: (**a**) external coating; (**b**) incorporation in the mixture at fresh state.

The evaluation of the properties of wood bio-concrete modified by silane–siloxane involved three treatment methods. The first method entailed the application of a water-based solution containing 5% silane concentration on the surface of the bio-concrete samples (Figure 2a). This surface protection, named external coating (WBC-EC), was applied in two coats with a total consumption of 13.7 mL/m^2^, as recommended by the manufacturer of the water repellent. The second method, based on Zhu et al. [19], involved the incorporation of a 1% concentration of silane emulsion (consumption of 7.66 kg/m^3^) during the production process of the bio-concrete (WBC-SIL) as an internal protection element (Figure 2b). The third method combined internal and external protection, incorporating silane into the bio-concrete mixture and applying the surface silane treatment at the same concentrations as defined in the previous methods (WBC-SIL+EC). Untreated bio-concretes were also produced as a reference (WBC-REF).

### 2.4. Cementitious Paste Test Procedures 

Silane–siloxane can be used as an internal protection element when it is incorporated into the mixture during the production process of concrete, which can result in microstructural changes in the cementitious paste. Therefore, this study examined the rheology and hydration of the cement paste used for producing bio-concrete with 1% silane relative in relation to the mass of the cementitious materials. A water-to-binder ratio of 0.4 was selected and the amount of silane introduced into the mixture was adjusted by reducing the amount of water. Table 2 provides the material consumption (in kg/m^3^) for producing the pastes studied.

#### 2.4.1. Rheological Tests 

The rheological tests were conducted using a Brookfield HD DV-III Ultra viscometer equipped with a four-bladed Vane spindle (V-75) with diameter of 8.03 mm. The paste-mixing procedure took place in a 5 L mortar mixer and involved four stages: (i) mixing cementitious materials for 1 min, (ii) progressive addition of water, (iii) low-speed (140 rpm) mixing for 2 min, and (iv) high-speed (285 rpm) mixing for 1 min. After the paste production, a volume of 150 mL was transferred to a glass beaker with a diameter of 56.3 mm. The elapsed time from water addition to the start of the test was approximately 8 min for all samples. Tests were performed in triplicate.

The shear rate protocol proposed by Tinoco et al. [31] was employed to obtain the flow curves. From this test, several parameters were determined. The static yield stress ($\tau_{0,s}$) was determined as the maximum stress value observed in the shear stress versus shear time curve. The dynamic yield stress ($\tau_{0}$) and viscosity (μ) were obtained by fitting the Bingham rheological model (Equation (1)) to the descending ramps of the flow curves.

(1) $$\tau =\tau_{0}+\mu \dot{\gamma }$$

where,

τ: shear stress (Pa);

$\tau_{0}$: dynamic yield stress (Pa);

μ: viscosity (Pa.s);

$\dot{\gamma }$: shear rate (s^−1^).

#### 2.4.2. Isothermal Calorimetry

To investigate the influence of silane–siloxane incorporation on the hydration kinetics of the paste, isothermal calorimetry tests were performed. The tests were conducted under isothermal conditions using the TAM Air equipment manufactured by TA Instruments, with a constant temperature of 25 °C. The paste production involved mixing and homogenizing the dry cementitious materials in a glass beaker, followed by the addition of water, with a total mixing time of around 3 min. Subsequently, approximately 5 g of the mixtures were placed into ampoules, which were sealed and placed in the isothermal calorimeter. The ampoules were introduced into the calorimeter approximately 6 min after water addition to the mixture. The tests were conducted for 5 days (120 h).

#### 2.4.3. Thermogravimetric Analysis

Thermogravimetric analysis (TGA) was performed on the pastes at 28 days of age using the SDT Q600 TGA/DTA/DSC simultaneous instrument from TA Instruments (New Castle, DE, USA). The analysis was carried out under the following experimental conditions: (i) an initial heating rate of 10 °C/min, starting from 23 °C and reaching 35 °C, followed by a one-hour isotherm at this temperature to facilitate sample drying and removal of uncombined water; (ii) subsequent heating at 10 °C/min until reaching a final test temperature of 1000 °C; (iii) a carrier gas of nitrogen with a flow rate of 100 mL/min; and (iv) utilization of an open alumina crucible.

### 2.5. Wood Bio-Concrete Test Procedures

#### 2.5.1. Flow Table and Entrained Air Tests

To assess the properties of the bio-concrete at fresh state, two tests were conducted: the flow table test and the entrained air content test. The flow table test was conducted in accordance with Brazilian standard NBR 13276 [32] and the entrained air content test was performed using adaptations of Brazilian standard NBR 16887 [33]. Both the tests were performed in triplicate.

#### 2.5.2. Compressive Strength 

The uniaxial compressive strength tests were conducted in accordance with the guidelines specified by the Brazilian standard NBR 5739 [34], and for the determination of the modulus of elasticity, the procedures prescribed in NBR 8522-1 [35] were followed. For each treatment configuration, including that of the reference specimens, five cylindrical specimens measuring 50 mm × 100 mm (diameter × height) were evaluated at 28 days. The tests were performed using a servo-controlled press, specifically the Shimadzu model-1000 kN, with a displacement rate of 0.3 mm/min. Longitudinal displacements were measured using a pair of LVDT (Linear Variable Differential Transformer) transducers from Controls (Liscate, Milan, Italy) that were attached to the samples.

#### 2.5.3. Capillary Water Absorption

The capillary water absorption of the bio-concrete was evaluated using a method proposed by RILEM (International Union of Laboratories and Experts in Construction Materials, Systems, and Structures). Specifically, the method follows the protocol in progress, denoted as TC HDB 275.

To conduct the test, the samples were dried in a 60 °C oven for 72 h. Five cylindrical specimens (50 mm × 100 mm) were used for each treatment. In order to ensure one-dimensional water transport, the lateral surfaces of the specimens were sealed using two layers of metallic tape. The bottom part of the samples were then immersed in water at a depth of 5 mm at specific time intervals, as follows: 1, 3, 5, 10, 15, and 30 min, 1, 2, 3, 4, 5, 6, 24, and 48 h. The capillary water absorption was determined by periodically weighing the specimens at the designated time intervals. 

## 3. Results and Discussion

### 3.1. Cementitious Paste

#### 3.1.1. Rheology 

**Figure 3:**
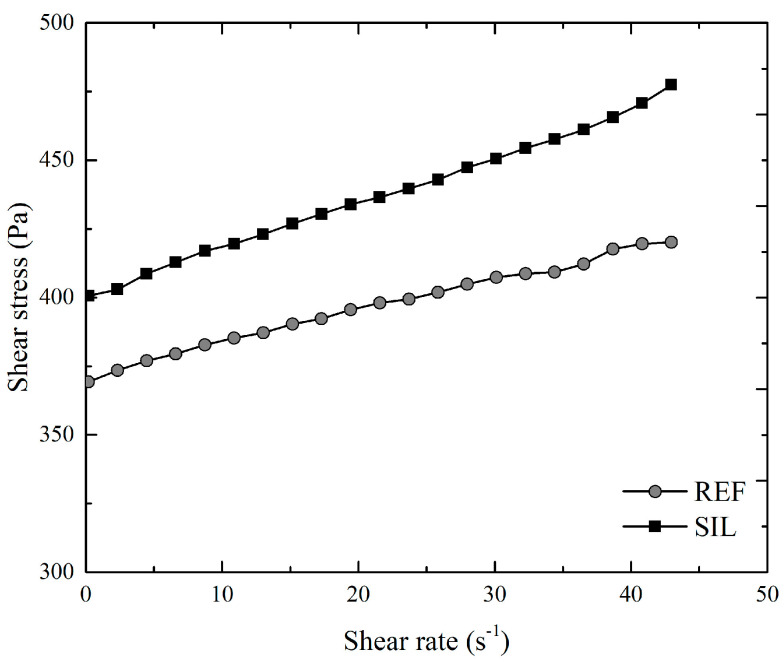
Flow curves obtained of the pastes.

Figure 3 shows the descending ramps of the flow curves obtained for the pastes, comparing those with and without silane incorporation. The flow curves exhibit an increase in shear stress with higher shear rates. Table 3 presents the rheological parameters derived from the tests. The data indicate that the incorporation of silane in the cementitious paste leads to increases in all rheological parameters. Specifically, there is approximately an 8% increase in the dynamic yield stress and a 40% increase in viscosity. This behavior can be attributed to the formation of a gel-film on the cement particles and hydrated products in the silane-modified paste. The gel-film is known to reduce the rate of ion diffusion in the early stages, potentially leading to increased viscosity in the mixture, as explained by Chen et al. [36].

The findings reported by Wang et al. [37] support the observed increase in the viscosity of cement pastes when incorporating graphene oxide and increasing silane concentration in the mixtures. Additionally, Yan et al. [38] reported an increase in the maximum shear stress with increasing silane addition up to a dosage limit of 2%. This indicates that the presence of silane facilitates the better engagement of the cementitious paste on the surface of the aggregates, resulting in a more compact structure.

#### 3.1.2. Isothermal Calorimetry 

**Figure 4:**
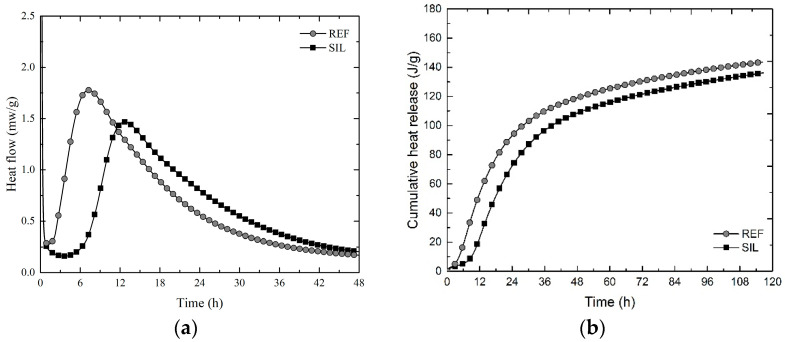
(**a**) Heat evolution curves; (**b**) cumulative heat release of the cementitious pastes REF and SIL.

The heat flow and cumulative heat release curves obtained in the isothermal calorimetry test are shown in Figure 4. The heat flow curves are depicted for a testing duration of up to 48 h. This time interval was chosen because all samples had completed a 24 h deceleration period.

From Figure 4a, it is observed that both REF and SIL heat flow curves showed the four stages of hydration: pre-induction, induction, acceleration, and deceleration. However, the incorporation of 1% silane in the mixture caused a delay in cement hydration. For SIL, the end of the induction period occurred at approximately 6.8 h, and the acceleration peak is observed at approximately 12.7 h, which is nearly 6 h after the reference.

The retarding effect of silane on cement hydration can be attributed to the adsorption and reaction of silane on the surfaceof cement hydration products. The adsorption of polymer molecules on these products impairs the hydration process, and the coupling reaction of silane with cement hydration products can further enhance the adsorption and lead to a delay in hydration [39]. Chen et al. [36] also note that silanes play a retarding role in cement paste at very early ages, with the proposed mechanism involving the formation of a gel-like barrier due to the attractive forces between intermediate products of silane hydrolysis and hydrated cement products. 

It is generally observed that the cumulative heat release in cement pastes containing silanes and silane derivatives is either lower than or similar to that of the control paste in the first 72 h [39]. The findings from this research support this proposition. Figure 4b depicts a slight reduction in the hydration heat of the SIL treatment, with a total accumulated heat value at the end of the test of 136.21 J/g. In comparison, REF exhibited a final value of 143.57 J/g.

#### 3.1.3. Thermogravimetric Analysis

**Figure 5:**
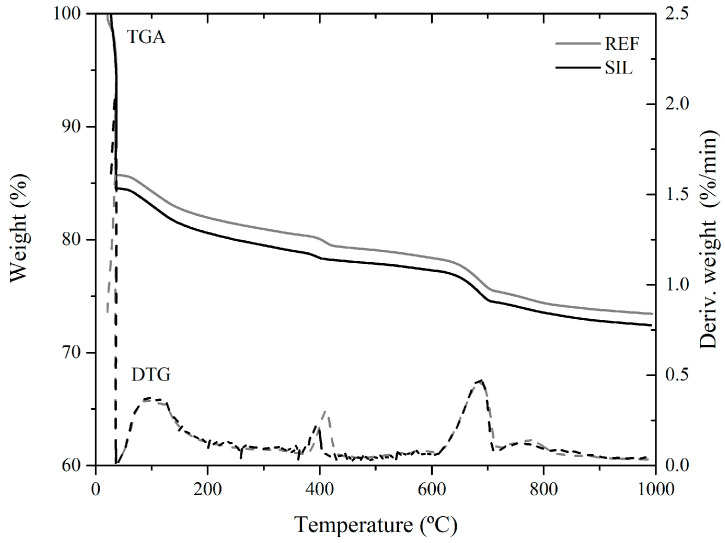
TGA–DTG curves of REF and SIL pastes.

TGA and DTG curves obtained in the test for both REF and SIL pastes (Figure 5) exhibit typical reactions that occur in cement-based pastes [40,41]. From DTG curves, three main peaks are observed for both cases: (i) The first peak, occurring up to approximately 395 °C, is associated with the dehydration of C-S-H (calcium-silicate-hydrate), ettringite (AFt and AFm phases), and aluminate phases present in the cementitious paste; (ii) the second peak, observed between 395 °C and 580 °C, corresponds to the dehydroxylation of calcium hydroxide (CH); and (iii) the third peak, observed at 580 °C and 780 °C, refers to the decomposition of amorphous and crystalline calcium carbonate (CaCO_3_).

The main difference observed between SIL and REF pastes lies in the peak corresponding to the dehydroxylation of calcium hydroxide (CH). In the SIL paste, the peak intensity for CH dehydroxylation is reduced compared to the REF paste, suggesting that a portion of the CH was consumed.

This result is in agreement with that determined by Chen et al. [36], who observed that the CH content of pastes produced with silane reduced by about 5% compared to that of a control paste. The explanation provided by Chen et al. [36] for this reduction in CH content involves the hydrolysis and polycondensation of silane in the presence of water, a process that may be accelerated in a cement solution with a high pH value. This hydrolysis and polycondensation of silane can lead to the formation of a structure similar to ${SiO}_{2}$, which is then capable of reacting with CH through the following reaction:

$${SiO}_{2}+CH\to C-S-H$$

Indeed, the small difference between the calcium hydroxide (CH) content in the SIL paste and the REF paste can be attributed to the incorporation of a relatively low silane content. It is important to consider that the efficiency of silane polymerization and the subsequent formation of a structure similar to SiO_2_ may depend on several factors including the silane dosage, the reactivity of the specific silane used, and the reaction conditions.

### 3.2. Wood Bio-Concrete

#### 3.2.1. Consistency Index, Entrained Air, and Bulk Density

Table 4 provides the results of the consistency index and entrained air content for wood bio-concrete with and without silane incorporation. The inclusion of the water repellent leads to a densification of the paste, resulting in an increase in its viscosity. This change is evident in the consistency index of the bio-concrete, which shows a decrease in this property of the composite. Despite the reduction in the consistency index, it was still feasible to cast the specimens solely by using mechanical vibration.

As observed in the reduction of the consistency index of the bio-concrete with the silane incorporation, a decrease in the content of air incorporated in the mixture was expected. With the addition of 1% silane, the composite produced had an air content value 51% lower than the reference value.

The average bulk density values of WBC-REF and WBC-SIL, at 28 days of age, were 1181 kg/m^3^ and 1247 kg/m^3^, respectively. The incorporation of silane in the mixture resulted in a reduction of the bio-concrete spreading compared to the reference, leading to increased density in the hardened state with just a 5% increment. This difference in density at the hardened state is directly correlated with the entrained air content in the fresh state (Table 4). The WBC-SIL mixture had a 5.9%-lower air content value compared to the WBC-REF mixture. Koohestani [42] reported that the addition of silane coupling agents as additives in cement-based materials could allow them to act as dispersing agents, leading to improved matrix densification.

WBC-EC and WBC-SIL+EC showed average values of bulk density of 1183 kg/m^3^ and 1246 kg/m^3^, respectively. These results suggest that the use of silane as a coating did not cause a significant change in the density of the bio-concrete samples. This observation is supported by the T-TEST statistical analysis conducted for the variables WBC-REF and WBC-EC, and for WBC-SIL and WBC-SIL+EC, assuming equivalent variances and a test significance level of 0.05 (5%). The statistical analysis did not reveal any significant difference in density between these samples. Based on that, it can be concluded that the application of silane as a coating does not lead to an increase in the density of the bio-concrete composite.

#### 3.2.2. Compressive Strength

**Figure 6:**
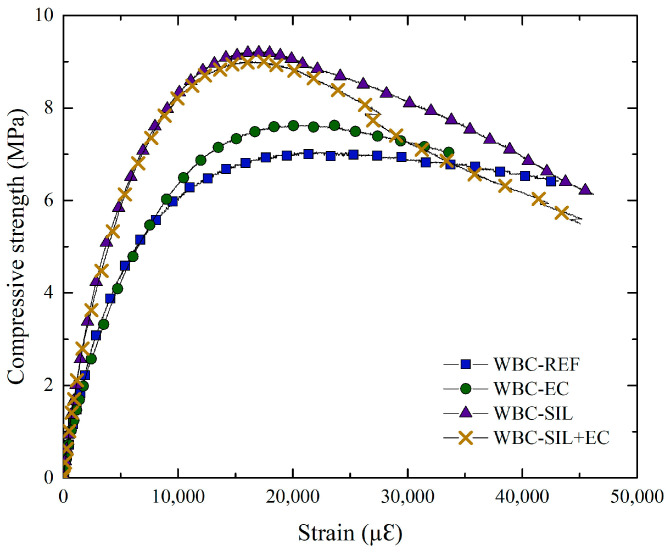
Compressive stress–strain behavior of the bio-concretes.

Figure 6 shows the typical stress -strain curves of bio-concrete with different silane treatments. The curves exhibit a common pattern, characterized by an initial linear region followed by non-linearity until reaching the peak stress and, finally, a post-peak behavior with significant deformation capacity. This behavior is attributed to the energy absorption promoted by the wood bio-aggregates present in the bio-concrete. Bio-aggregates are principally composed of cellulose, hemicellulose, and lignin [43]. Cellulose is the major component, and its chains are organized in the form of microfibrils, with their arrangement determining the deformation capacity of the plant particles [44].

The comparison of stress–strain curves between WBC-REF and WBC-EC, as well as between WBC-SIL and WBC-SIL+EC, indicating similar behavior, suggests that the use of external coating (WBC-EC) did not significantly alter the mechanical properties of the bio-concrete composite. However, when comparing the stress–strain curves of bio-concrete with and without incorporated silane, more noticeable differences become apparent. The incorporation of silane resulted in an increase in the compressive strength and modulus of elasticity for both WBC-SIL and WBC-SIL+EC compared to WBC-REF and WBC-EC. This enhancement in mechanical properties can be attributed to the effect of silane on the cementitious matrix and its interaction with the wood bio-aggregates, which led to improved bonding and densification overall. Moreover, in the post-peak region, a significant reduction of up to 33% in compressive strength for a strain of 40,000 με is observed for WBC-SIL+EC, while WBC-REF and WBC-EC show minimal stress reduction with increasing strain.

Table 5 provides the results of the maximum compressive strength achieved and the modulus of elasticity for the bio-concretes studied. The data indicate that the incorporation of silane in the mixture resulted in a significant increase of approximately 24% in compressive strength and 94% in the modulus of elasticity, comparing WBC-REF and WBC-SIL. This enhancement in mechanical properties is associated with the decrease in the entrained air content of the bio-concrete caused by the incorporation of silane. Chen et al. [36] observed a better mechanical performance of silane-modified pastes compared to pure pastes at 28 days due to lower porosity and a higher degree of hydration. Based on the results obtained, it can be inferred that the external coating of silane did not change the compressive strength of the bio-concretes. Otherwise, the addition of silane in the mixtures improved the mechanical properties.

#### 3.2.3. Capillary Water Absorption

**Figure 7:**
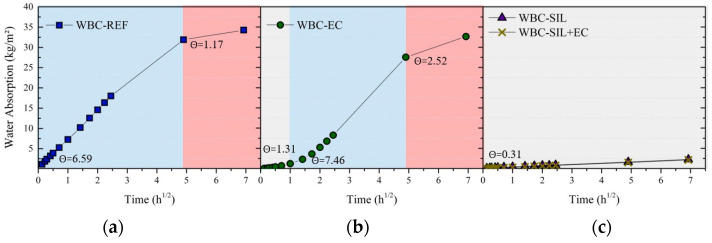
Capillary water absorption curves of the bio-concretes (**a**) WBC-REF, (**b**) WBC-EC, (**c**) WBC-SIL, and WBC-SIL+EC.

**Figure 8:**
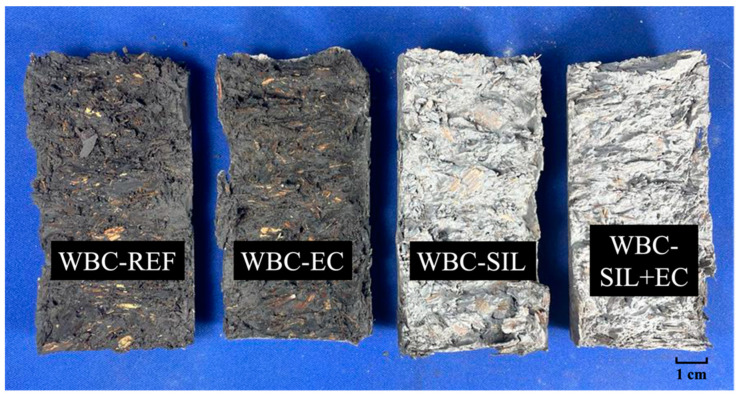
Capillary rise of water in bio-concrete samples.

Figure 7 illustrates the capillary water absorption curves of the studied bio-concretes. The untreated bio-concrete (WBC-REF) demonstrated a continuous and significant increase in water absorption capacity over time. The WBC-REF curve (Figure 7a) shows two sections with different absorption rates (Θ): (i) the first was characterized by rapid water absorption up to 24 h of testing, with a value of Θ = 6.59 kg/m^2^·h^1/2^; (ii) and the second Θ decreased to 1.17 kg/m^2^·h^1/2^. By the end of the 48 h test, WBC-REF absorbed 34.3 kg/m^2^ of water. The high water absorption capacity observed in WBC-REF can be attributed to the combination of a high volume fraction of bio-aggregate (45%) and a porous cementitious matrix. The presence of a large volume of bio-aggregate in the composite contributes to its porous nature, allowing for a high potential for water absorption. Moreover, the capillary rise effect, as illustrated in Figure 8, facilitates the transport of water to the top of the sample, leading to the observed high water absorption over time.

The capillary water absorption behavior of WBC-EC showed a non-linear and time-varying pattern. As depicted in Figure 7b, the silane external coating effectively prevents rapid water absorption by the composite during the first hour of the test (Θ = 1.31 kg/m^2^·h^1/2^). During this period, the absorption value remains at approximately 1.2 kg/m^2^, which is significantly lower (approximately 6 times) than that observed in WBC-REF. On the other hand, after one hour of testing, a change in behavior occurs and the material begins to exhibit greater water absorption up to 24 h of testing (Θ = 7.46 kg/m^2^·h^1/2^). This change is attributed to the continuous presence of a water layer at the bottom of the specimens. As this water layer persists, it gradually overcomes the thin layer of silane, allowing for the transport of water to occur more rapidly within the bio-concrete. After 24 h of testing, the absorption rate drops to 2.52 kg/m^2^·h^1/2^.

The data from the capillary water absorption test confirm that the silane external coating (WBC-EC) is indeed efficient in preventing rapid water absorption during the initial hours of testing. As indicated in the results, after 6 h of testing, the absorption value for WBC-EC is 8.3 kg/m^2^, which is significantly lower compared to that for WBC-REF (34.3 kg/m^2^). However, over an extended period of 48 h of testing in this study, the effectiveness of the external coating diminished and the water absorption capacity increased substantially, reducing the final absorption value by only about 5% in relation to WBC-REF. The visual observation of the water depth in the specimens further confirms this behavior. Both WBC-EC and WBC-REF show similar water depth, reaching the top of the specimen (Figure 8) after 48 h of testing.

The results obtained for WBC-SIL (Figure 7c) demonstrate its excellent water-repellent properties and high resistance to water absorption. The curve for WBC-SIL in the capillary water absorption test exhibits a distinct behavior compared to WBC-REF and WBC-EC, especially in terms of slope, which indicates a significantly lower absorption rate (Θ = 0.31 kg/m^2^·h^1/2^). The incorporation of silane into the bio-concrete mixture effectively creates a hydrophobic matrix, preventing the entry of water and capillary rise within the composite and improving its durability. The absorption value of WBC-SIL after 48 h of testing was only 2.3 kg/m^2^, which is even lower than the value obtained for WBC-REF in a 3 min test (2.4 kg/m^2^). This significant reduction in water absorption demonstrates the excellent performance of WBC-SIL when applied as a water repellent agent in the cementitious matrix while still in the fresh state.

The WBC-SIL+EC combination showed no change in capillary absorption behavior in relation to WBC-SIL, with both curves practically overlapping and showing the same absorption rate. The statistical analysis using T-TESTwith equivalent variances further supports this observation as it indicates that there is no statistically significant difference between the absorption values of WBC-SIL and WBC-SIL+EC after 48 h of testing.

Table 6 presents the absorption values at all study times for each type of treatment.

Figure 8 provides a visual representation of a transversal section of the different bio-concrete samples (WBC-REF, WBC-EC, WBC-SIL, and WBC-SIL+EC) immediately after the end of the capillary water absorption test. As previously mentioned, for the WBC-REF and WBC-EC samples, the capillary rise of water reached the top of the specimens. On the other hand, for the WBC-SIL and WBC-SIL+EC specimens, there was no apparent rise of water at the height of the test specimens. This visual observation confirms the highly effective water-repellent properties of the bio-concrete with internal silane incorporation.

## 4. Concluding Remarks

Based on the findings reported in this experimental investigation, the following conclusions can be drawn:

(i) The incorporation of 1% silane in the cementitious paste increased the rheological parameters, delayed hydration by approximately 6 h compared to the reference, and slightly decreased the heat of hydration in 5%.
(ii) Wood bio-concrete with silane incorporation (WBC-SIL) showed an 8% lower consistency index and the entrained air content in fresh state was reduced by 51% in comparison to WBC-REF.
(iii) The use of external coating (WBC-EC) did not alter the mechanical properties of the composite. On the other hand, the addition of silane in the mixture (WBC-SIL and WBC-SIL+EC) caused an increase of approximately 24% in compressive strength and 94% in the modulus of elasticity.
(iv) WBC-EC treatment was effective in capillary water absorption only in the first hour of the test (with the absorption rate being reduced by 81% compared to WBC-REF in the same period) while the addition of silane to the mixture (WBC-SIL and WBC-SIL+EC) showed excellent performance, with a reduction in the absorption rate up to 95% compared to WBC-REF, preventing the entry of water and capillary rise within the composite and improving its durability.

## 5. Study Limitations and Future Research

The main limitations of this study was the use of a single type of bio-aggregate and the lack of standards for testing bio-based materials. For future directions, the authors suggest carrying out more mechanical tests (such as splitting), tests on full-size blocks, and the evaluation of the durability to natural aging of these materials in addition to modeling the results obtained.

## Figures and Tables

**Table 1:** Mix proportion of WBC (kg/m^3^).

	BA	PC	RHA	FA	Wh	Wc	CC
WBC	238.50	302.41	168.01	201.61	268.81	166.95	13.44

**Table 2:** Mix proportion of cementitious pastes (kg/m^3^).

Pastes	PC	RHA	FA	Wh	Silane
REF	549.83	305.46	366.56	476.52	-
SIL	549.83	305.46	366.56	464.12	12.22

**Table 3:** Rheological parameters (coefficient of variation in parentheses).

Pastes	$\tau_{0,s}$ (Pa)	$\tau_{0}$ (Pa)	μ (Pa.s)	R^2^
REF	992.96 (16.4%)	371.84 (1.3%)	1.15 (8.4%)	0.994
SIL	1479.04 (12.2%)	402.95 (3.5%)	1.61 (6.9%)	0.996

**Table 4:** Consistency index and incorporated air content results of WBC (coefficient of variation in parentheses).

Wood Bio-Concretes	Consistency Index (mm)	Entrained Air Content (%)
WBC-REF	185 (5.4%)	11.5 (1.2%)
WBC-SIL	170 (2.9%)	5.6 (3.6%)

**Table 5:** Compressive strength and modulus of elasticity of each of the bio-concretes (coefficient of variation in parentheses).

Wood Bio-Concretes	Compressive Strength (MPa)	Modulus of Elasticity (GPa)
WBC-REF	7.54 (3.84%)	0.85 (6.79%)
WBC-EC	7.51 (4.37%)	0.88 (10.94%)
WBC-SIL	9.35 (4.15%)	1.65 (14.42%)
WBC-SIL+EC	9.27 (3.45%)	1.53 (14.54%)

**Table 6:** Water absorption values in kg/m^2^ (coefficient of variation in parentheses).

Time	WBC-REF	WBC-EC	WBC-SIL	WBC-SIL+EC
1 min	1.15 (29.0%)	0.06 (12.9%)	0.09 (26.7%)	0.06 (49.4%)
3 min	1.88 (24.7%)	0.11 (8.2%)	0.14 (27.5%)	0.08 (28.1%)
5 min	2.41 (21.9%)	0.18 (7.4%)	0.17 (26.5%)	0.09 (27.6%)
10 min	3.22 (19.6%)	0.29 (37.0%)	0.23 (21.5%)	0.14 (17.8%)
15 min	3.89 (18.5%)	0.40 (43.8%)	0.25 (25.8%)	0.15 (19.7%)
30 min	5.28 (17.0%)	0.69 (49.0%)	0.34 (23.5%)	0.22 (14.8%)
1 h	7.27 (15.8%)	1.19 (48.2%)	0.42 (24.2%)	0.29 (11.4%)
2 h	10.20 (16.3%)	2.28 (46.0%)	0.57 (22.1%)	0.41 (13.3%)
3 h	12.54 (17.1%)	3.62 (37.7%)	0.68 (20.5%)	0.55 (10.8%)
4 h	14.59 (18.1%)	5.23 (29.2%)	0.77 (19.2%)	0.64 (12.5%)
5 h	16.34 (18.4%)	6.76 (24.4%)	0.80 (18.9%)	0.68 (12.9%)
6 h	17.99 (18.4%)	8.25 (21.8%)	0.89 (17.4%)	0.76 (14.2%)
24 h	31.87 (9.9%)	27.55 (15.8%)	1.61 (11.1%)	1.49 (9.9%)
48 h	34.25 (3.2%)	32.67 (8.6%)	2.25 (8.3%)	2.14 (7.4%)

## Data Availability

Not applicable.
